# Developing the best model for telemonitoring triage: experiences and insights

**Published:** 2012-06-15

**Authors:** Matthew Rutter, David Barrett

**Affiliations:** Ernst and Young LLP, UK; University of Hull, UK

**Keywords:** teleheath, triage, telemonitoring

## Abstract

**Introduction:**

The demographic, clinical and financial pressures faced by healthcare providers have led to the adoption of innovative methods for delivering care, such as telehealth. If telehealth can be considered as an ‘umbrella’ term, encompassing all applications of technology to remotely support healthcare and promote well-being, then ‘telemonitoring’ can be viewed as a specific application linked to remote monitoring of physiological measures, signs and symptoms. A maturing clinical evidence base—including two positive Cochrane Reviews (Inglis et al., 2010; McLean et al., 2011)—coupled with increased awareness and improving technology, has led to an increase in telemonitoring adoption over the past few years. However, though the number of telemonitoring deployments is rising, there remains uncertainty in regards to the most effective model of delivery. This presentation focuses specifically on the different models that exist for triaging and responding to the data generated by patients receiving telemonitoring.

**Aims and objectives:**

1. To outline the broad service model common to telemonitoring deployments (input, process, and output)
2. To highlight the different approaches to generating, triaging and responding to clinical and technical alerts
3. To discuss the strengths and weaknesses of different approaches to triage and response
4. To share real-world experiences of developing and implementing different models of triage and response

**Results:**

The presenters have been involved with telemonitoring deployments utilising a range of different service models. The presentation will describe this spectrum of models, ranging from ‘closed-loop’ systems where triage and response is largely automated, through to centralised telemonitoring teams providing expert clinical triage. Each model has its own strengths and weaknesses. For example, automated systems will reduce costs, but may lessen the ‘human’ elements of care often valued by patients. Conversely, a centralised team of nurses offering clinical triage and response is likely to give added value to a telemonitoring service, but at the cost of substantial financial outlay. Many models of delivery sit within these polar opposites, and the relative strengths and weaknesses of key examples will be discussed. In addition to a theoretical overview, the presentation will also allow for the discussion of real-world experiences of developing and operationalizing different models of triage and response. Examples from Yorkshire and the Humber will be discussed, and delegates will be asked to share their own experiences and perspectives. A specific example from a GP practice in Yorkshire will also highlight how models of triage and response can be tailored to dovetail with existing processes.

**Conclusion:**

The successful adoption of telemonitoring at scale requires a clear understanding of the optimum service model for a specific deployment. This presentation will provide a pragmatic overview of the models available for triage and response, allowing delegates to consider the best structure for telemonitoring deployment in their own localities.

